# Instructed Task Demands and Utilization of Action Effect Anticipation

**DOI:** 10.3389/fpsyg.2012.00578

**Published:** 2012-12-28

**Authors:** Robert Gaschler, Dieter Nattkemper

**Affiliations:** ^1^Department of Psychology, Humboldt-Universität zu BerlinBerlin, Germany; ^2^Department of Psychology, Universität Koblenz-LandauLandau, Germany; ^3^Berlin Research Center Image Knowledge GestaltungBerlin, Germany

**Keywords:** action effect, effect anticipation, stimulus based, intention based, instruction manipulation

## Abstract

Automatic acquisition of action effect associations may serve as a parsimonious account of how people acquire the basis for intentionally controlled action. However, recent research suggests that learning or the expression of action effect links might depend on whether task demands impose either a stimulus based mode of action control or an intention based action control mode. In the current study we develop a paradigm that allows the mode of action control to be varied via instructions while keeping stimuli identical. Participants were to respond to the location of a cloud of dots. Their actions were followed by predictable visual effects, either consistently congruent or incongruent with the location of the action. In Experiment 1, a displaced new cloud of random dots was presented as a spatial action effect. In Experiment 2 an arrow was presented as effect with a pointing direction congruent or incongruent to the response position. The location of the stimulus in the reference frame was easy to detect in some of the trials while the location of the cloud of dots was completely ambiguous in others. The instruction manipulation targeted the latter trials, suggesting to one group of participants to freely choose a key in a difficult trial, while asking another group to react to their spontaneous impression in the event of a difficult stimulus. In this way, we aimed at rendering actions either as stimulus driven or internally generated. By this we could investigate how effect anticipation changed with practice depending on action mode. We employed the impact of action effect compatibility on speed and choice of action as a measure for action effect anticipation. Our results suggest that action effect associations can be acquired when instructions suggest stimulus based action control or intention based action control. Instructions aiming at the mode of task processing can influence when and how action effect links influence behavior.

## Introduction

Human actions are controlled by one of two principles. On the one hand, people *react* to stimuli in the environment that are associated with a response based on a known and practiced mapping (e.g., stopping at a red traffic light). On the other hand, people *choose* actions according to their current goals when prompted to do so (e.g., sign with branching arrows). Red color can lead to a direct abrupt halt in the case of a traffic light, but may serve as a cue to choose between stopping to enjoy the view vs. continuing a journey in the case of a meadow with poppy flowers.

While the ideomotor approach (e.g., James, 1890; Greenwald, 1970; Prinz, 1987; see Shin et al., 2010) generally holds that actions are triggered by their anticipated perceptual consequences, recent research (Herwig et al., 2007; Herwig and Waszak, 2009) has suggested that this might be especially pronounced in endogenously triggered actions (see also Umbach et al., 2012 and Kemper et al., 2012). In simple tasks requiring that one of two keys is pressed with tones as action effects, participants readily formed associations between actions and action effects – if they were freely choosing actions when prompted. However, in contrast to past findings with explicit instructions (e.g., Kunde, 2001) or more complicated mappings (cf. Ziessler et al., 2004) no action effect learning was evident when participants were responding based on stimulus discrimination. This challenging finding warrants further exploration for several reasons. Conditions differed in stimulus material (i.e., free choice prompt vs. stimulus to be discriminated) *and* instruction (i.e., discriminative response required vs. free choice required). Free choice prompts vs. stimuli in choice reaction tasks differed with regard to the visual features (i.e., variability in the stimulus vs. homogeneity in the prompt).

We suggest that in many situations free choice vs. forced choice is a matter of how a situation is interpreted. This interpretation can be set up by instructions and other constraints and can quickly shift in character from one second to the next one. On a trip with young children, a sign of a restroom can, depending on the instruction the children provide, either trigger immediate action or, alternatively, trigger considerations on whether or not the facilities should be used in order to avoid later hurry. A branch in the road with a short and slow vs. a fast and long route might lead to a spontaneous choice on some days while on others we are told to hurry and thus, the choice aspect of the branching seems less pronounced. Situations prompting a free choice vs. those demanding a certain reaction might occur in close succession. It is conceivable that boosting action effect associations in a free choice situation transfers to a more structured forced choice situation. Pfister et al. (2011) have made a similar suggestion. They proposed that an intention based mode of action adopted in a free choice task might be maintained in a forced choice task. Without such a spillover effect of formerly established action mode, action effect associations might remain ineffective and thus undetected in a choice reaction task that is performed by strong reliance on stimulus based rather than goal directed processing.

In the current study the measurement of action effect associations employed the backward compatibility between (a) action and (b) the anticipated consequences. A response that is expected to be followed by a matching effect can be executed faster than a response that likely will lead to a non-matching effect. If anticipation of action effects (i.e., the activation of effect codes) is a constitutional part of action planning, then effects should have a backward influence on action (cf. Kunde et al., 2004). Specifically, the compatibility of the current action with the anticipated action effect should influence the speed with which the action is executed. For instance, pressing a left key that will predictably lead to a visual action effect on the left should be faster compared to a left key press that will lead to an action effect on the right. For instance, similar to the current study, Pfister et al., 2010, Experiment 2) presented participants with a mix of free choice and forced choice trials. They used the reaction time (RT) of responses that predictably led to an incompatible vs. to a compatible action effect as a measure of the influence of action effect anticipation on behavior. On forced choice trials, participants saw an arrow pointing left or pointing right as a stimulus demanding a left or right key press. On free choice trials an exclamation mark demanded a free response choice. In addition, Pfister et al. (2010) used cues at the beginning of each trial to signal whether the action effects would be incompatible (effect was a box on the screen on the side opposite to the response) or compatible (effect was a box on the same side) or neutral (box in the middle). Results showed that the anticipation of an incompatible action effect led to slower RTs than the anticipation of a neutral or compatible effect. Notably, this was the case for free choice as well as forced choice trials when they were mixed (Experiment 2). In the blocked version (in Experiment 1), however, only the free choice trials led to an impact of action effect compatibility on RT.

Different from the design of the current study, acquisition of action effect associations has often been assessed in terms of compatibility effects in a test phase after a substantial amount of learning trials (cf. Greenwald, 1970; Elsner and Hommel, 2001; Herwig et al., 2007; Herwig and Waszak, 2009, 2012). First participants perform a learning phase in which actions are predictably followed by specific effects (auditory or visual). Next, in a test phase participants are presented with the former action effects as stimuli. In one version (e.g., Elsner and Hommel, 2001; Maes, 2006; Hoffmann et al., 2009) they are to freely choose a response whenever one of the former action effects is presented while using the same responses as in the learning phase. The dependent measure is the proportion of free choices that follows the association of action and effect that was present in the learning phase. In another version (e.g., Hommel et al., 2003; Herwig et al., 2007; Herwig and Waszak, 2009) participants are prescribed which key to press for either of the former action effects once these are presented as stimuli in the test phase. In this case, the dependent measure is the RT advantage for a setup in which the assignment of keys to former action effect matches rather than mismatches the experiences from the learning phase.

Test phases are generally applied after substantial exposure to the contingency between actions and effects. Therefore, the dynamics of how the impact of action effect compatibility on performance evolves with practice are difficult to assess – unless variations to the length of the learning phase or multiple test phases are used (cf. Wolfensteller and Ruge, 2011). These dynamics are especially relevant in the light of a recent discussion, which suggests that stimulus based action control mode vs. intention based action control mode lead to differences in the *expression* of action effect knowledge rather than its acquisition (Ansorge, 2002; Pfister et al., 2010, 2011). It is conceivable that action effect associations are acquired in a learning phase with stimulus based action control (cf. Hommel, 1996; Ziessler et al., 2004) just as in a learning phase with intention based action control (e.g., Herwig and Waszak, 2009), but are not expressed in a later test phase if action control is stimulus based. Pfister and colleagues suggested that the mode of action control might shift slowly. It is therefore possible that when participants perform under stimulus based mode of action control at the end of the learning phase, they might transfer the according mode of action control to the test phase. Therefore, action effect associations might not influence behavior.

In choice reaction tasks, action control might not be purely stimulus based, but instead include effect anticipation. Before reaching a high level of proficiency in a choice reaction task, participants are likely to use anticipated action effects *and* stimulus response (S-R) links to control behavior (cf. Hazeltine, 2002; Band et al., 2009; Gaschler et al., 2012). Only at the end of a sufficiently long practice phase, strong S-R links might lead to a situation where stimulus based action control dominates over intention based control (compare also Ackerman and Woltz, 1994). It might be difficult to predict whether and when the change in mode of action control takes place. This suggests continuous assessment of action effect anticipation rather than assessment with a single test phase. Apart from the amount of practice it may for instance depend on the saliency of action effects as well as on the size of the set of actions, stimuli, and effects whether or not the stimulus based mode of action control becomes dominant during a choice reaction task or not. Work successfully employing forced choice tasks for providing participants with action effect links has usually used a setup with four keys and at least four action effects (cf. Ziessler et al., 2004; Nattkemper et al., 2010). The work directly contrasting action effect learning under stimulus based vs. intention based action control (Herwig et al., 2007; Herwig and Waszak, 2009) has used smaller sets. In the latter case, stimulus based mode of action control might have become dominant by the end of the acquisition phase. If it would have been tested, action effect associations may have influenced RT at the beginning of a learning phase when task demands were still high.

**Figure 1:**
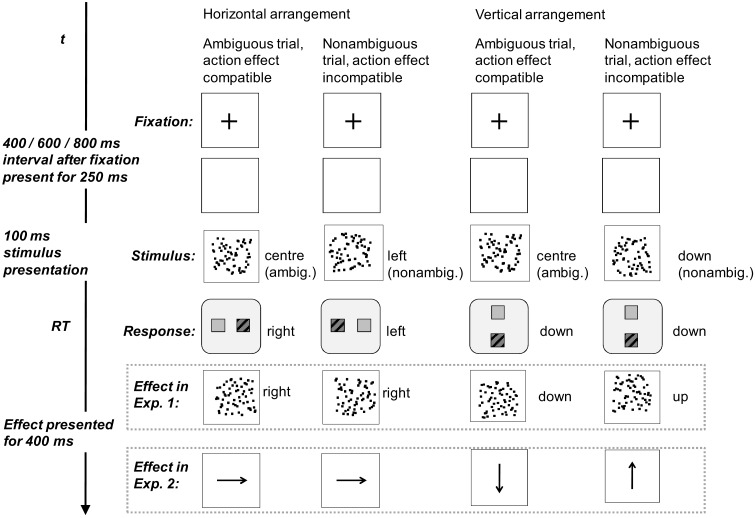
**Example stimuli and time-line for Experiment 1**. In Experiment 2, arrows were used as action effects instead of clouds of dots.

The present work targets how, depending on the mode of action control, practice affects the acquisition and expression of action effect associations. Rather than employing a design involving a learning and a test phase, we used a setup similar to Pfister et al., 2010, see above). Via instructions we aimed at manipulating whether participants assumed an action vs. reaction focus. The task contained 1/3rd of trials in which stimuli were ambiguous with respect to the relevant feature stimulus position. In these trials, a random cloud of dots was placed exactly in the middle of a reference frame – rather than at one of its borders. For the two instruction conditions, identical stimuli (random clouds of dots) were either referred to as prompts for free choices or as to be discriminated stimuli. In the forced choice instruction condition, stimulus based action control was suggested as instructions asked for fast key presses spontaneously conveying the first impression of the position of a cloud within the reference frame (left vs. right; or up vs. down – depending on balancing scheme; compare Figure 1). In the free choice instruction condition, intention based action control was fostered as participants were told in advance that the position of the clouds of dots was often indiscriminate and in such cases freely chosen spontaneous key presses were the action to be taken. The ambiguous trials, in which the position of the cloud could not be objectively discriminated, were intermixed with trials with a clear placement of the cloud of dots in the reference frame. We assumed that, depending on instructions concerning the ambiguous trials, participants would either adopt an intention based or a stimulus driven mode of action control. According to the suggestion of Pfister et al. (2011), this mode of action should also influence the processing of non-ambiguous stimuli. Thereby, instruction can be expected to modulate the expression of action effect associations in RTs in trials with discriminable and indiscriminable stimuli.

So far, an experimental paradigm has been lacking that allows the choice character of a task involving action effect learning to be flexibly varied. Flexibility might either result from varying stimulus discriminability on a continuum or, alternatively, from varying instructions while keeping stimuli identical. For instance, depending on instructions, identical stimuli might be either processed as free choice prompts or as difficult stimuli in a discrimination paradigm. Conceivably, it may even be possible to gradually vary the extent to which a task is processed as a discrimination task vs. a free choice task by varying stimulus discriminability between (a) clearly discriminable and (b) perfectly ambiguous.

Here we report the first steps for exploring the space of experimental manipulations for such a paradigm. Gradual variations of stimulus discriminability have been systematically applied in research on perceptual grouping principles (e.g., grouping by proximity or by similarity in Kubovy and Van den Berg, 2008). The probability of perceiving one rather than the other version of a multi-stable ambiguous stimulus gradually increased with increasing proximity or similarity. Changes in reports varied gradually between extreme ends of stimulus discriminability (i.e., between stimuli with one dominant percept vs. stimuli with two equally likely percepts). The authors refer to their approach as phenomenological psychophysics. There are no objectively (in)correct answers as it is the participant’s task to report what they perceive in a multi-stable stimulus. Participants can only respond incorrectly in the sense that they do not truthfully report their percept.

It is an open question whether or not both desired options for experimental manipulation are effective at the same time – (a) instruction for free choice vs. for stimulus discrimination and (b) variation in stimulus discriminability. Conceivably, instructions overrule objective discriminability. If participants are told to truthfully report their percepts they might perform in forced choice mode even if stimuli are objectively perfectly ambiguous. Conversely, participants who are told to freely press any key in the case of difficult to discriminate stimuli may transfer this free choice mode of action control even to easily discriminable stimuli. For instance, Pfister et al. (2010) have suggested that once an intention based mode of action control is established through free choice trials, this mode of action control might also transfer to trials with an imperative stimulus. This suggests that the same easily discriminable stimuli might be processed in one or the other mode of action control depending on whether the ambiguous stimuli in the surrounding trials are interpreted as free choice prompts or as difficult to discriminate stimuli.

We tested the impact of instructions concerning the mode of action control on action effect anticipation in two experiments. In Experiment 1 we used a setup that employed clouds of dots both as stimuli/free choice prompts and as action effects. Action effects were highly similar to the stimuli in order to ensure that participants could not avoid processing the visual action effects. In Experiment 2 we used arrows as action effects instead.

## Materials and Methods

### Participants

Fifty-eight university students from Berlin (38 female; mean age 23 years, SD = 5.6) took part in *Experiment 1* and were paid €6. They were randomly assigned to the free choice vs. forced choice instruction condition (*N* = 29 for both conditions). Due to hardware failure, the data of one participant from the free choice condition were lost. Seventy university students from Berlin took part in *Experiment 2* (57 female; mean age 23 years, SD = 4.0) and randomly assigned to either instruction condition. Three participants were excluded due to procedural problems, leaving 35 participants in the free choice condition and 32 in the forced choice condition.

### Materials

Experiment 1 and 2 were identical except in the action effects used (see below). Stimuli and response effects consisted of random clouds of dots presented as single white pixels in a 100 by 100 white reference frame on black background (Figure 1). They were presented on a 17 ″ CRT screen at a resolution of 800 × 600 pixels. As explained below, we used (a) blocks with a horizontal arrangement of stimuli, responses and effects and (b) blocks with a vertical arrangement. Each trial started with a fixation cross displayed for 250 ms before the beginning of the randomly selected stimulus onset asynchrony (SOA) of 400, 600, or 800 ms. The fixation cross was replaced by an empty reference frame. The stimulus, a randomly generated cloud of dots was then presented for 100 ms and was afterward replaced by an empty reference frame. In Experiment 1, another cloud of dots was presented as the action effect (presented for 400 ms) just 30 ms after the participant pressed a key. In Experiment 2 a white 2 cm long, bold arrow was presented as action effect on each trial instead of a cloud of dots. The arrow could point left, right, upward, or downward.

Stimulus clouds consisted of 64 white dots. They were generated by pairing 64 *X* coordinates with *Y* coordinate randomly without replacement (one dot every column). Clouds presented as action effects (Experiment 1) were generated by the identical mechanism, but shifted to the left or to the right by 6 pixels within the reference frame. One third of the stimulus clouds were presented centrally (ambiguous trials) and the rest were evenly split between either left or right and up or down (non-ambiguous trials).

For each participant, the experiment consisted of two parts of three blocks each. Action effect compatibility as well as axis for stimuli, responses and effects was consistent in each half. In the first three vs. the last three blocks of the task we used a horizontal vs. a vertical variant for placing stimuli, effects and for the responses. In addition, action effects were either consistently compatible or incompatible in the first vs. last three blocks. The order of compatibility conditions and the order in which the task with the horizontal vs. vertical axis were performed, were balanced across participants. Depending on which axis was used, participants either placed the left or right index fingers on the keys 4 and 6 (left and right) or 8 and 2 (up and down) on the number pad of a regular keyboard. Keys were covered by white stickers of 1.5 cm diameter and the number pad was placed centrally in front of the monitor. Within either the first vs. last three blocks of the task just two keys and action effects (left and right *or* up and down) were relevant. In this way, we avoided that participants would experience both compatible and incompatible action effects for the same keys. Past work has suggested that apart from external action effects, characteristics of the keys are dominant with respect to the coding of the responses (i.e., coding by key position; compare Hoffmann et al., 2009; Gaschler et al., 2012). Therefore, it seemed crucial to change the keys for blocks with compatible vs. incompatible action effects. Consider for instance, a participant who was presented with left and right clouds of dots as stimuli. This person executed left vs. right key presses and was presented with compatibly placed clouds of dots as action effects in the first three blocks. In the last three blocks, this person executed up vs. down key presses and experienced incompatible down vs. up action effects.

### Procedure

In the computerized instructions, the trial structure was explained to the participants and they were instructed to use their index fingers to press a key when the first cloud appeared, while the second cloud (action effect Experiment 1) or arrow (action effect in Experiment 2) should not be followed by a key press. Participants were told to press the key (left vs. right or up vs. down) that corresponded to the spatial location of the cloud within the reference frame. They were informed that in many trials it would be very difficult to determine the location. Our experimental conditions differed with respect to how participants were instructed to proceed in these trials. Participants in the *forced choice condition* were asked to spontaneously and quickly select either key depending on their impression of the location of the cloud. It was stressed that key presses could be false only in the sense that they might not correspond to the impression (cf. Kubovy et al., 1998). Thus participants in the forced choice condition were instructed that they were selecting key presses based on the stimulus – no matter whether this stimulus was easy or hard to categorize as left or right (or up vs. down). In contrast, in the *free choice condition* participants were instructed to freely press either left vs. right (or upper vs. lower key) whenever the location of the cloud was hard to determine. If the location was clear, participants were to select the key to press accordingly.

Following the instruction, participants completed three blocks of 120 trials each. Before action effect compatibility (and axis of stimuli, responses, and effects) was reversed for the last three blocks, participants completed an intermediate task of 120 choice reaction trials without programed action effects. Participants were randomly presented either letter D or H centrally on the screen and had to press the key corresponding to the letter on the keyboard with their index fingers. The intermediate block served as a baseline for comparing general speed in the two instruction groups and as a buffer between the assessment of speed with compatible vs. incompatible action effects. After each block, participants received feedback concerning their mean latency. The experiment was completed within approximately 60 min.

## Results

### Practice-related changes in how action effect compatibility affected RTs

**Figure 2:**
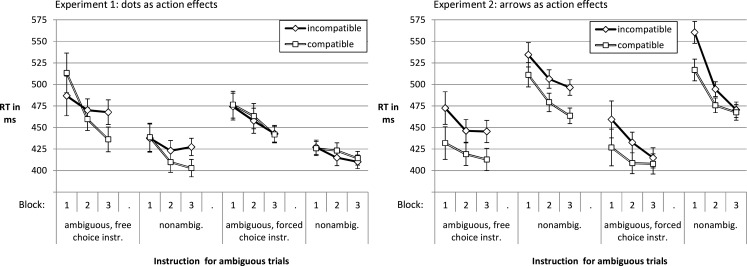
**Reaction times per block and condition in Experiments 1 and 2**. The error bars in this and all other graphs reflect ± the standard error of the compatibility effect at each factor level of the other factors.

Figure 2 displays the mean RTs per block and condition averaged over participants. RTs decreased with practice for ambiguous and non-ambiguous trials, both in the free choice and in the forced choice condition. In *Experiment 1*, compatibility of response effects increasingly influenced RT over the course of the three blocks in the free choice condition while it had no influence in the forced choice condition. These impressions were confirmed by a 2 (compatibility of effects: compatible vs. incompatible) × 2 (ambiguity of stimuli: ambiguous vs. non-ambiguous) × 3 (practice: Block 1–3) × 2 (instruction: free choice vs. forced choice) mixed analysis of variance (ANOVA). Here and elsewhere we applied Greenhouse–Geisser correction if warranted. There was a main effect of practice, *F*(1.7, 93.22) = 20.87, *p* < 0.001, $\eta_{p}^{2}=0.28$, and one of ambiguity, *F*(1, 55) = 89.72, *p* < 0.001, $\eta_{p}^{2}=0.62$. In both groups of participants, non-ambiguous trials evoked faster key presses than ambiguous ones. Given the tendency of reverse effects of compatibility late vs. early in training, there was no main effect of compatibility, *F* < 1. Most relevant for the experimental hypothesis, action effect compatibility increasingly influenced response speed with practice in the free choice instruction condition, while no such dynamic was evident in the forced choice condition. This was reflected by an interaction of compatibility, practice, and instruction condition, *F*(1.75, 95.99) = 3.32, *p* = 0.047, $\eta_{p}^{2}=0.06$. Furthermore, the speedup with practice was more pronounced in ambiguous as compared to the non-ambiguous trials, *F*(1.99, 109.18) = 18.7, *p* < 0.001, $\eta_{p}^{2}=0.25$, for the interaction. As this was first and foremost based on the compatible trials, there was also a triple interaction of compatibility, ambiguity, and practice, *F*(1.77, 97.22) = 3.65, *p* = 0.035, $\eta_{p}^{2}=0.06$. The interaction of compatibility and practice was not significant, *F*(1.75, 95.99) = 2.76, *p* = 0.075, $\eta_{p}^{2}=0.05$ (other *F*s < 1.8).

In deviation to Experiment 1, there was an effect of action effect compatibility from the first block onward in *Experiment 2*, both in the free choice as well as in the forced choice condition. Interestingly, there was a tendency for the quickly established effect to decrease with practice in the forced choice condition while it was stable in the free choice condition. In variation to Experiment 1, RTs were lower for ambiguous as compared to non-ambiguous trials. The ANOVA documented a main effect of ambiguity, *F*(1, 65) = 161.98, *p* < 0.001, $\eta_{p}^{2}=0.71$, one of practice, *F*(1.59, 103.39) = 51.85, *p* < 0.001, $\eta_{p}^{2}=0.44$, and one of compatibility, *F*(1, 65) = 13.49, *p* < 0.001, $\eta_{p}^{2}=0.17$. The decrease of the impact of ambiguity on RT with practice was reflected in an interaction with ambiguity, *F*(1.88, 122.41) = 25.58, *p* < 0.001, $\eta_{p}^{2}=0.28$. As this reduction was more pronounced for the participants in the forced choice as opposed to those in the free choice condition, there was a triple interaction of instruction, ambiguity, and practice, *F*(1.88, 122.41) = 3.7, *p* = 0.03, $\eta_{p}^{2}=0.05$. This differential reduction in RT seemed to be driven by the incompatible rather than by the compatible trials. However, the interaction of compatibility, instruction, ambiguity, and practice was not significant, *F*(1.55, 100.76) = 2.58, *p* = 0.094, $\eta_{p}^{2}=0.04$ (other *F*s < 2.3).

Our results on response speed in the additional choice reaction task without artificial action effects (reacting with key D vs. H to letter D vs. H) rule out sampling biases between the two instruction conditions. In *Experiment 1*, participants assigned to the free choice (*M* = 394.6 ms) and the forced choice (*M* = 394.2 ms) condition in the main task performed at identical speed in this extra task (*M* = 393.1 ms vs. *M* = 383.5 ms for *Experiment 2*).

### Additional analyses

Experiment 1 and 2 only differed with respect to the action effects. However, the pattern of RT results reported above was rather different in a setup in which clouds of dots were used as stimuli *and* effects (Experiment 1) or only as stimuli (Experiment 2, arrows as effects). Aiming at first suggestions for explanations for this surprising difference, additional analyses were performed on choice and RTs. We investigated how choice in ambiguous trials differed after free choice vs. forced choice instruction, whether action effect compatibility influenced response choice, and how the switching between ambiguous and non-ambiguous trials affected performance. To foreshadow, the analyses document that action effect compatibility and instruction (forced choice vs. free choice) influenced choice and RTs in both experiments. While in Experiment 1, effects were found either in RTs or in choice, the effects were less mixed and more straightforward in Experiment 2. Conceivably, the specific feature of Experiment 1 that was responsible for the difference was the high overlap between stimuli and effects. We assume that participants faced difficulties in differentiating stimuli and effects. Likely, they chose responses such that the overlap was reduced or were slowed down.

We analyzed response choice in order to check whether participants were attending and processing the location of the clouds. One has to bear in mind that participants were to either choose freely or respond according to their impression of the stimulus. They received no feedback. Stimuli were presented only briefly and the position of the non-ambiguous clouds was only slightly shifted away from the center of the reference frame (compare Figure 1). Thus, a ceiling effect in stimulus-following trials with non-ambiguous stimuli was not to be expected. In the following we will first present summary analyses of the choice data focusing on the extent to which both possible responses were used with balanced frequency in a block. We will then proceed to analyses focusing on sequencing aspects of stimuli, effects, and responses and report to which extent the position of the cloud of dots in the reference frame determined responses in non-ambiguous trials.

#### Compatibility and instructions driving deviations from balanced response frequencies

**Figure 3:**
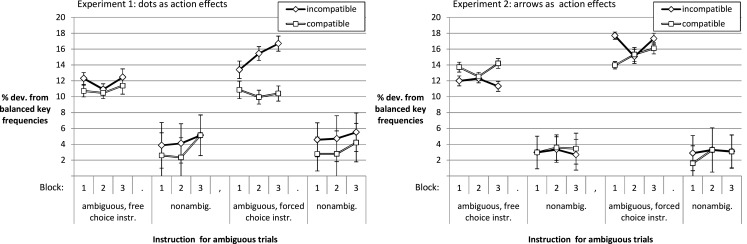
**Deviation from balanced response frequencies**.

We analyzed idiosyncratic response biases in order to (a) check the validity of our instruction manipulation and to (b) test the impact of compatibility. Concerning the latter issue, it has been suggested that generation of random response patterns is resource demanding and that deviations from randomness can increase under challenging task demands (cf. Jahanshahi et al., 2006). Thus, deviations from randomness (in our case indicated by deviation from balanced frequencies) might be larger in blocks with incompatible response effects. With respect to potential challenges to the validity of our free choice instruction, one should consider that many participants might not invest the effort to choose a response trial-by-trial. Rather, they may press one key “per default” on the ambiguous trials. As we cannot know in advance which of the two keys this might be, we calculated the deviation from balanced response frequencies. For each participant, block, compatibility, and ambiguity condition we determined the (absolute) deviation from pressing both keys with 50% frequency. As Figure 3 shows, this bias was higher in the ambiguous as compared to the non-ambiguous trials. It was more pronounced in blocks with incompatible rather than compatible action effects. Furthermore, it increased with practice. For *Experiment 1* the 2 (compatibility of effects: compatible vs. incompatible) × 2 (ambiguity of stimuli: ambiguous vs. non-ambiguous) × 3 (practice: Block 1–3) × 2 (instruction: free choice vs. forced choice) ANOVA yielded a main effect of compatibility, *F*(1, 55) = 7.68, *p* = 0.008, $\eta_{p}^{2}=0.12$, a main effect of stimulus discriminability, *F*(1, 55) = 170.27, *p* < 0.001, $\eta_{p}^{2}=0.76$, and one of practice, *F*(1.98, 109.11) = 3.55, *p* = 0.033, $\eta_{p}^{2}=0.06$ (other *F*s < 2.08).

For *Experiment 2* the same ANOVA documented a main effect of ambiguity, *F*(1, 65) = 190.58, *p* < 0.001, $\eta_{p}^{2}=0.75$. The impact of ambiguity on deviation from balanced response frequencies was *larger* in the forced choice than in the free choice condition, *F*(1, 65) = 4.66, *p* = 0.035, $\eta_{p}^{2}=0.07$, for the interaction of instruction condition and ambiguity. This means, participants who were instructed to respond to difficult stimuli spontaneously according to their impression of the stimulus were more biased toward one response than the participants who were instructed to freely choose a key in case of difficult stimuli. There was no main effect of compatibility (*F* < 1), but there was a tendency toward an interaction of instruction condition and compatibility, *F*(1, 65) = 3.06, *p* = 0.085, $\eta_{p}^{2}=0.05$. While in the forced choice condition the blocks with incompatible action effects led to Δ = 1% more deviation from balance than compatible blocks, this was reversed for the free choice instruction condition (Δ = 0.98%). Furthermore, there was a tendency toward a main effect of instruction condition as the deviation from balanced response frequencies was 1.47% higher overall in the forced choice compared to the free choice condition, *F*(1, 65) = 2.86, *p* = 0.095, $\eta_{p}^{2}=0.04$ (other *F*s < 1.46).

In summary, the results suggest that stereotyped responding does not seem to be a problem that threatens the validity of the free choice instruction condition. The deviation from balanced frequencies was not larger in the free choice as compared to the forced choice condition – rather the opposite was true. Furthermore, we observed a larger deviation from randomness in the incompatible blocks (i.e., the more demanding condition, cf. Jahanshahi et al., 2006). In addition, the choice data document an impact of action effect compatibility even at the beginning of Experiment 1, and instructions (free choice vs. forced choice) modulated choice behavior.

#### Proportion of response repetitions affected by compatibility

Response repetitions in free choice trials have been suggested as a measure of feature binding (e.g., Janczyk et al., 2012a). As action effect compatibility was administered in a blocked manner, a repetition of the response led to a repetition of the action effect as well. Binding between actions and effects might foster response repetitions: when a participant pressed a key, an effect was presented. The effect just presented might have activated the response just given to elicit this effect.

**Figure 4:**
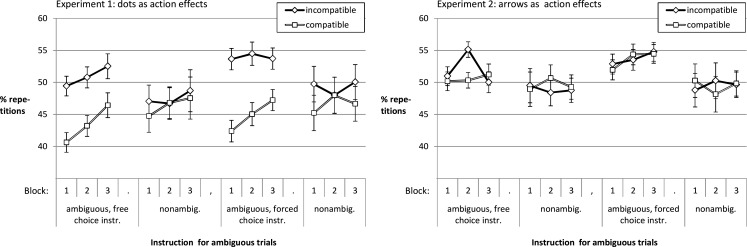
**Time course for proportion of response repetitions**.

Figure 4 shows that the proportion of response repetitions depended on practice as well as on stimulus ambiguity and action effect compatibility. For *Experiment 1* the ANOVA yielded a main effect of compatibility, *F*(1, 55) = 28.25, *p* < 0.001, $\eta_{p}^{2}=0.34$. Incompatible blocks led to more repetitions as compared to compatible blocks. The proportion of repetitions increased with practice, *F*(2, 104.52) = 3.73, *p* = 0.029, $\eta_{p}^{2}=0.06$. Furthermore, the impact of compatibility on the rate of response repetitions was more pronounced in ambiguous rather than non-ambiguous trials, *F*(1, 55) = 23.98, *p* < 0.001, $\eta_{p}^{2}=0.3$, for the interaction effect (other *F*s < 1.76).

The ANOVA for *Experiment 2* only confirmed that there were more response repetitions in ambiguous as compared to non-ambiguous trials, *F*(1, 65) = 13.55, *p* < 0.001, $\eta_{p}^{2}=0.17$. Somewhat surprisingly, there was an interaction of compatibility, ambiguity, practice, and instruction condition apparently reflecting the peak in response repetitions in Block 2 of the incompatible ambiguous trials in the free choice condition, *F*(2, 129.99) = 4.19, *p* = 0.017, $\eta_{p}^{2}=0.06$ (other *F*s < 1.57).

Taken together the results suggest that the overlap between stimuli and effects in Experiment 1 led participants to avoid response repetitions. They were apparently doing so especially in ambiguous trials, in blocks with compatible action effects, and at the beginning of the experiment. One can speculate that perceiving the effect on the side of the response led to a contrast effect that fostered response alternation to ambiguous stimuli. If, for instance, the participant pressed the left key and saw a cloud of dots shifted left, it might have become apparent that this effect (as opposed to ambiguous stimuli) was *clearly* positioned on the left. This might have evoked the tendency to try the opposite response on the next ambiguous stimulus. We will follow up on this in the next section.

#### Instructions and past trial ambiguity affecting proportion of response repetitions

**Figure 5:**
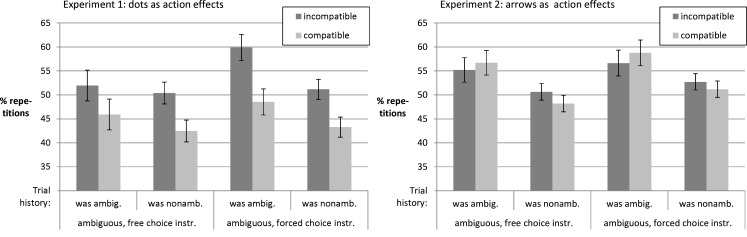
**Proportion of response repetitions in ambiguous trials depending on trial history and action effect compatibility**.

In a second step we focused on ambiguous trials and the trials immediately preceding them. We separately calculated the proportion of response repetitions separately for ambiguous trials following non-ambiguous or ambiguous ones. The data were subjected to a 2 (compatibility of effects: compatible vs. incompatible) × 2 (ambiguity of stimuli *in previous trial*: was ambiguous vs. was non-ambiguous) × 2 (instruction: free choice vs. forced choice) ANOVA. Mirroring the analysis in the last section, participants in *Experiment 1* were more likely to repeat the response of the previous trial in blocks with incompatible rather than compatible action effects, *F*(1, 55) = 29.57, *p* < 0.001, $\eta_{p}^{2}=0.35$. Figure 5 further suggests that participants were more likely to repeat the response if the stimulus in the prior trial was ambiguous rather than non-ambiguous, *F*(1, 55) = 20.8, *p* < 0.001, $\eta_{p}^{2}=0.27$. Further, there was an interaction of instruction condition and ambiguity in the previous trial, *F*(1, 55) = 20.8, *p* = 0.036, $\eta_{p}^{2}=0.08$ (other *F*s < 1.7). The impact of past trial ambiguity on chance to repeat the response in the current ambiguous trial was higher in the condition with the forced choice instructions as compared to the condition with the free choice instructions. Participants for whom instructions had suggested to discriminate the spatial position of an ambiguously placed cloud of dots were more likely to repeat the response as compared to those participants who were instructed to freely press any key.

In *Experiment 2* we obtained a similar increase of the rate of response repetitions in ambiguous trials following ambiguous rather than non-ambiguous trials, *F*(1, 65) = 23.18, *p* < 0.001, $\eta_{p}^{2}=0.26$. Compatibility had no effect (*F* < 1). Furthermore, there was a tendency toward an interaction of compatibility and ambiguity in the previous trial, *F*(1, 65) = 3.23, *p* = 0.077, $\eta_{p}^{2}=0.05$. If the previous trial was non-ambiguous, participants were by *M* = 1.99% more likely to repeat the response in conditions with incompatible as compared to compatible response effects. However, if the previous trial was ambiguous, the compatibility effect was reversed (*M* = 1.81% less response repetitions in incompatible as compared to compatible blocks; other *F*s < 1).

In summary, supporting the validity of our instruction manipulation, the impact of characteristics of past and present stimuli on response choice was larger in the forced choice instruction condition than in the free choice condition. While the feature binding account (cf. Herwig and Waszak, 2012; Janczyk et al., 2012a) would have predicted that the rate of response repetitions should be highest when effects match stimuli and stimuli repeat, we found the highest rate of response repetition in blocks with incompatible action effects and especially so if the preceding stimulus had been ambiguous as well. It is conceivable, that in Experiment 1, incompatible action effects as well as response alternations helped to disentangle stimuli (to be reacted to) from effects (no reaction required) which overruled any potential positive effects of action effect binding on response repetition.

#### Instruction, compatibility, and practice affecting stimulus-following in non-ambiguous trials

Next we scrutinized whether participants followed the non-ambiguous stimuli in their response choices. We were interested in whether free choice instructions concerning the ambiguous stimuli were also affecting the non-ambiguous ones. Furthermore, practice-related changes in stimulus-following and the impact of action effect compatibility were of interest.

**Figure 6:**
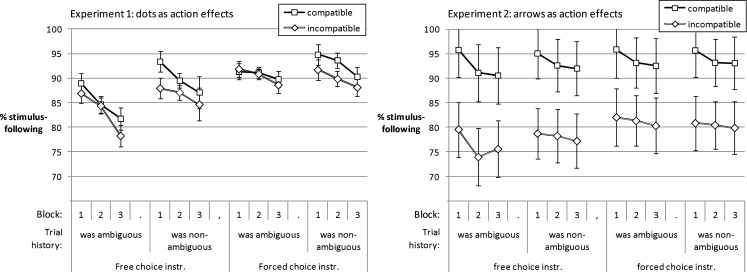
**Time course of proportion of trials with non-ambiguous stimuli in which participants followed the stimulus in their response choice**.

As suggested by Figure 6, the rate of stimulus-following in non-ambiguous trials was higher in compatible as compared to incompatible blocks and deteriorated with practice. For *Experiment 1* the 2 (compatibility of effects: compatible vs. incompatible) × 2 (ambiguity of stimuli *in previous trial*: ambiguous vs. non-ambiguous) × 3 (practice: Block 1–3) × 2 (instruction: free choice vs. forced choice) ANOVA yielded a main effect of instruction condition, *F*(1, 55) = 4.51, *p* = 0.038, $\eta_{p}^{2}=0.08$. Participants in the forced choice instruction condition more often based their responses on the position of the cloud of dots than participants in the free choice condition. Further, there was a main effect of compatibility, *F*(1, 55) = 6.25, *p* = 0.015, $\eta_{p}^{2}=0.1$. Participants more often followed the response suggested by the stimulus in blocks with compatible rather than in incompatible effects. In the former case the response was in line with the upcoming effect. The main effect of ambiguity of the previous stimulus, *F*(1, 55) = 23.11, *p* < 0.001, $\eta_{p}^{2}=0.3$, reflected that participants less often responded according to the non-ambiguous stimulus if the previous stimulus had been ambiguous rather than non-ambiguous. This was especially pronounced in the free choice rather than in the forced choice condition, *F*(1, 55) = 10.45, *p* = 0.002, $\eta_{p}^{2}=0.16$, for the interaction of instruction condition and prior ambiguity. Stimulus-following decreased with practice, *F*(1.76, 96.71) = 23.46, *p* < 0.001, $\eta_{p}^{2}=0.3$. There was a tendency of a qualification suggesting that the decline might be steeper in the free choice instruction condition rather than in the forced choice instruction condition. The interaction between instruction condition and practice was not significant, *F*(1.76, 96.71) = 2.52, *p* = 0.092, $\eta_{p}^{2}=0.04$.

Furthermore, the advantage of stimulus-following in compatible compared to incompatible blocks was more pronounced after non-ambiguous rather than after ambiguous trials, *F*(1, 55) = 7.22, *p* = 0.01, $\eta_{p}^{2}=0.12$, for the interaction. The decrease in stimulus-following with practice was strongest for trials after ambiguous ones in the free choice condition, *F*(1.75, 95.99) = 3.94, *p* = 0.028, $\eta_{p}^{2}=0.07$, for the interaction of prior ambiguity, practice, and instruction condition (other *F*s < 2.08).

Participants in *Experiment 2* also more often followed the stimulus in blocks with compatible rather than incompatible action effects, *F*(1, 65) = 14.4, *p* < 0.001, $\eta_{p}^{2}=0.18$, for the main effect of compatibility. Stimulus-following deteriorated with practice, *F*(1, 65) = 10.38, *p* < 0.001, $\eta_{p}^{2}=0.14$. The advantage of the forced choice instruction condition over the free choice condition in stimulus-following was 3.12% if the previous trial was ambiguous, but instruction conditions differed by only 1.55%, if the previous trial was non-ambiguous. This led to an interaction of instruction condition and prior ambiguity, *F*(1, 65) = 4.62, *p* = 0.035, $\eta_{p}^{2}=0.07$. In tendency, the decline in stimulus-following was steeper for trials following ambiguous rather than non-ambiguous trials. The interaction of prior ambiguity and practice was not significant, *F*(1.93, 125.18) = 2.74, *p* = 0.007, $\eta_{p}^{2}=0.04$ (other *F*s < 1.83). The graph shows that the standard error of the compatibility effect was much larger in Experiment 2 as compared to Experiment 1. This was because some of the participants in Experiment 2 were influenced by the compatibility of the action effects much more strongly than others. Apparently, incompatible arrows as action effects led them to reverse responses to the non-ambiguous stimuli in many trials. Thus, while the arrows as action effects were likely not to be confused with the stimuli, the response to the cloud of dots was nevertheless biased by the compatibility of the action effects.

Taken together, the validity of the instruction manipulation was again supported. Stimulus-following was stronger in the forced choice instruction condition than in the free choice condition – yet the impact of the stimulus position on response choice was substantial even in the latter condition. While there was an effect of action effect compatibility on stimulus-following in all conditions of Experiment 2, the compatibility effect in Experiment 1 depended upon the interaction of trial history and instruction.

#### Switching costs

On the one hand, mode of action control might show some inertia and transfer from ambiguous to non-ambiguous trials (cf. Pfister et al., 2010 and results reported above). On the other hand, instructions in the free choice condition might, in principle, have led to the configuration of two different task sets, one with stimulus based and one with intention based action control, with participants switching between these two task sets on a trial-by-trial basis. Switching between ambiguous and non-ambiguous stimuli might therefore have involved switch costs for participants in the free choice instruction condition. For participants in the forced choice instruction condition, such a switch was not suggested by the instructions as they were told to respond according to their impression of the stimulus in all trials. In order to obtain a full picture of events repeating vs. alternating from one trial to the next, we split the analysis for compatible vs. incompatible action effects.

**Figure 7:**
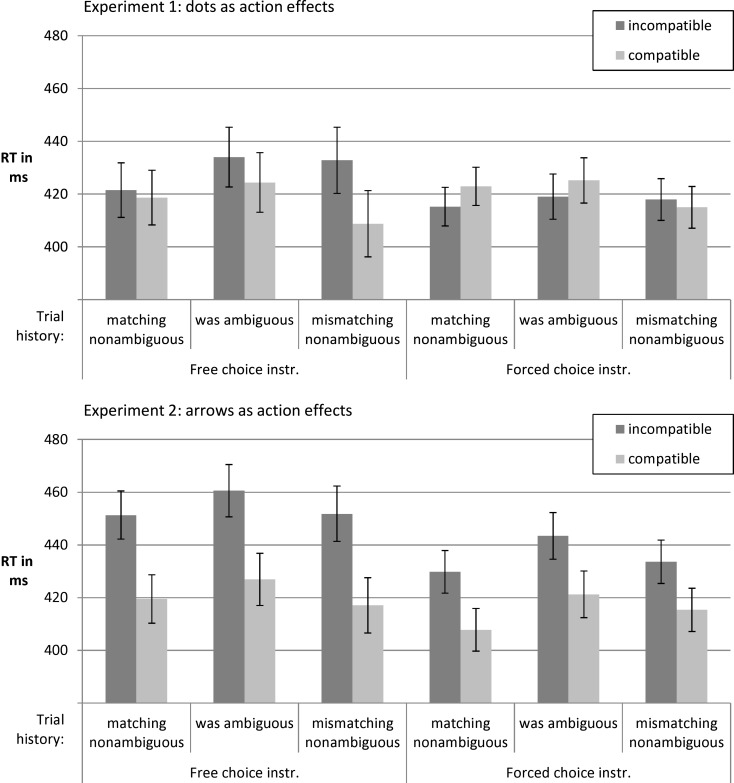
**RTs in trials with non-ambiguous stimuli depending on trial history and action effect compatibility**.

As shown in Figure 7, the impact of action effect compatibility on RT in *Experiment 1* depended on which type of trial was present before the current non-ambiguous trial. This seemed to be especially pronounced in the free choice instruction condition. Incompatible effects led to slowing especially if the past trial was a non-ambiguous trial with a stimulus different to the one in the current trial. In this case the (incompatible) action effect of the previous trial was highly similar to the stimulus in the current one. Conversely, participants were especially fast when they switched from one non-ambiguous stimulus and its compatible effect to the opposite stimulus and effect. It is conceivable that participants ran the risk of confusing effects and stimuli whenever effects were identical to upcoming stimuli and were therefore slowed. This account is speculative. It should be stressed that the analysis also showed that there was a substantial RT effect of action affect compatibility in some conditions in Experiment 1.

Apart from RT effects of action effect compatibility, we observed costs of switching from an ambiguous stimulus to a non-ambiguous one. This effect did not merely reflect costs of a stimulus alternation between subsequent trials. Rather, RTs after ambiguous trials were slower than both RTs after non-ambiguous trials with repeating vs. alternating stimuli. For *Experiment 1* the ANOVA showed a main effect of previous stimulus, *F*(1.89, 102.71) = 5.51, *p* = 0.005, $\eta_{p}^{2}=0.09$, as well as an interaction of compatibility and previous stimulus, *F*(1.99, 109.17) = 8.07, *p* = 0.001, $\eta_{p}^{2}=0.13$ (other *F*s < 1.48). In *Experiment 2* there was also a main effect of previous stimulus type, *F*(1.84, 119.64) = 10.13, *p* < 0.001, $\eta_{p}^{2}=0.14$. Furthermore, there was a main effect of compatibility, *F*(1, 65) = 18.44, *p* < 0.001, $\eta_{p}^{2}=0.22$ (other *F*s < 1).

We expected trial history to have a larger impact in the free choice as compared to the forced choice instruction condition. According to the instructions, task switching and the respective costs might have been involved in the former but not in the latter. In the free choice condition the instruction suggested different task sets for ambiguous (free choice) vs. non-ambiguous stimuli (respond according to stimulus) whereas in the forced choice condition the task was always to respond according to the impression that the stimulus produces. While the pattern of means in Figure 7 seems consistent with this view, the ANOVA did not confirm an instruction effect. Potentially, the higher RT following an ambiguous trial rather reflects aftereffects of adaptation to conflict or difficulty (e.g., Dreisbach and Fischer, 2011).

### Summary of additional analyses

Supporting the validity of the instruction manipulation, the impact of characteristics of past and present stimuli on response choice was larger in the forced choice as compared to the free choice instruction condition. Taken together, Experiments 1 and 2 show that whether action effect compatibility influences response speed and/or response choice is highly dependent upon context factors such as instruction (forced choice vs. free choice) and the specific combination of stimuli and effects employed in the experiment. The additional analyses provide important qualifications for the interpretation of the RT results initially presented. The impression might have been that action effect associations had to be acquired over the course of practice in Experiment 1 and that this acquisition took place only in the free choice condition. Response choice however, was affected by action effect compatibility, stimulus discriminability, and instruction already early in practice. The choice data strengthen the argument that the instructed task set modulates the impact of action effect compatibility on performance.

## Discussion

The present work aimed at developing an experimental paradigm that allows to flexibly vary the choice character of a task involving action effect learning. The role of intention based vs. stimulus based action control in the acquisition and/or expression of action effect associations is currently under debate (Herwig and Waszak, 2009, 2012; Pfister et al., 2011). We assumed that flexible experimental variation of mode of action control can either be granted by varying instruction while keeping the stimuli identical and/or by varying stimulus discriminability. Therefore, we designed a paradigm in which participants received visual stimuli, some of which were ambiguous. Clouds of random dots were either briefly presented shifted toward one boarder of a reference frame or were presented centrally. Responses were followed by predictable visual effects – clouds of dots shifted in position in Experiment 1, or arrows in Experiment 2. The influence of action effect anticipation on performance was assessed by comparing the RT for blocks in which response position and action effect mismatched (incompatible) vs. matched (compatible). In addition we analyzed which reaction participants chose. We targeted the mode of action control by an instruction manipulation. Instructions either suggested to the participants that difficult to discriminate stimuli should evoke a spontaneous key press (free choice condition) or a spontaneous reaction in accordance with the first impression that the stimulus leaves (forced choice condition). In line with Pfister et al. (2011) the reported findings suggest that action effect associations can be acquired when instructions suggest a stimulus based action control mode or an intention based action control mode. However, instructions moderated how action effect associations influenced performance.

### Instruction-induced action mode

Our results suggest that the instruction-induced mode of action control can determine how and when action effect compatibility influences task performance in conditions that only differ in instructions. For ambiguous as well as for non-ambiguous stimuli, the impact of action effect compatibility on RT and response choice depended upon what the instructions suggested concerning the difficult to discriminate trials. In Experiment 1, free choice instructions had the consequence that with practice action effect compatibility gained influence on RT. With forced choice instructions, there was no RT effect. However, analysis of response choice suggested that action effect compatibility influenced performance throughout practice in all instruction conditions. In Experiment 2, we observed effects of action effect compatibility on RT and on choice in all conditions from the first block onward. With practice, however, the RT effect of action effect compatibility vanished in the forced choice instruction condition.

We assume that instructions helped to establish or maintain stimulus based vs. intention based action control. While the impact of graded variations of stimulus discriminability on action mode remains to be explored for the future, the current data provide an example that instructions can overrule extreme cases of stimulus discriminability. Participants who were told to freely press any key in case of difficult to discriminate stimuli apparently transferred this free choice mode of action control even to easily discriminable stimuli. In a similar vein, Pfister et al. (2010) have suggested that once an intention based mode of action control is established due to free choice trials, this mode of action control might also transfer to trials with an imperative stimulus. In the present case, ambiguous and non-ambiguous trials were randomly intermixed. This might make such transfer likely (compare Experiment 2 of Pfister et al., 2010). Furthermore, the mixture ensured that participants paid attention to the discriminative characteristics of the stimulus for ambiguous and non-ambiguous trials alike.

At first glance the above considerations seem contradictory. On the one hand, instructions concerning trials in which the position of the cloud of dots was difficult to determine affected whether or not with practice an influence of action effect compatibility on RT was observed – both for ambiguous and for non-ambiguous trials. Therefore, the influence of instructions seemed to operate at a general level. On the other hand, response choice in non-ambiguous trials clearly showed that the response was determined by the stimulus in most trials. Apparently, action was intention based but at the same time influenced by the stimulus. This finding is in line with work from implicit sequence learning stressing that in choice reaction tasks, the processing and implicit learning of response goals can outweigh processing and learning concerning the stimuli – despite participants are generally reacting accurately to the stimuli (cf. Willingham et al., 2000; Ziessler and Nattkemper, 2001; Hazeltine, 2002; Abrahamse et al., 2010; Gaschler et al., 2012). These results can be reconciled by proposing some inertia to intentional action control. At least, before strong S-R links are established, action control can be intentional in choice reaction tasks (Ackerman and Woltz, 1994; Hommel, 2000). Interspersed free choice trials might counteract a shift from intention based action control to largely stimulus based performance that might otherwise take place with practice.

As a note of caution we have to admit that in the current study we aimed at manipulating mode of action control but lack a measure of mode of action control that is independent of the main dependent variables. A manipulation check probing the efficacy of instructions to establish one vs. the other task set has for instance been employed in Gaschler et al. (2012). It should be developed for the current purpose as well to avoid circularity of arguments. A further issue is the general *decrease* of stimulus-following in non-ambiguous trials with block of practice. It might contradict the idea that performance becomes less intention based and more stimulus based with practice. Alternatively, stimulus based processing may with practice be applied more homogeneously to most non-ambiguous and ambiguous trials. Lapses of attention might set in with practice and lead to a situation where in some trials neither stimuli nor effects are attended to.

### Stimuli resembling action effects

In Experiment 1 action effects were very similar to the stimuli. This similarity ensured that the visual action effects were attended to. However, action effect compatibility with effects similar to the stimuli might be considered as a special case. This case has previously been investigated with biological stimuli. For instance, Brass et al. (2000), studied compatibility between observed and executed finger movements. Future research should manipulate the nature of and the overlap between stimuli and effects more systematically. In the current study it is likely that at the same time we changed stimulus-effect overlap and effect saliency when we changed from clouds of dots as action effects (Experiment 1) to arrows (Experiment 2). We assume that in the beginning, when confronted with a novel task, the quickly acquired action effect associations could impact RT in Experiment 2, as there were no problems of overlap between stimuli and effects. Intention based control may have been effective early on, being partially substituted by stimulus based control with practice (cf. Ackerman and Woltz, 1994). This might have led to the reduction of the impact of action effect compatibility on RT in the forced choice instruction condition of Experiment 2. Future studies should explore potential tradeoffs between action effect compatibility affecting RT vs. choice.

The comparison of the experiments suggests that stimulus-effect compatibility is but one source that influences response choice. The impact of action effect compatibility on response choice was not restricted to the setup with overlapping stimuli and effects. With arrows as action effects (Experiment 2) stimulus-following for non-ambiguous stimuli was higher in blocks with compatible as compared to incompatible effects. Furthermore, comparing how the influence of action effect compatibility on RT changes with practice in Experiment 1 and 2 illustrates that results can critically depend upon when the assessment of action effect associations takes place and which measures are being considered. Compatibility had an influence on RT at the end of practice in the free choice condition for ambiguous and non-ambiguous trials in both experiments. Throughout practice, the forced choice condition showed no RT effect of action effect compatibility in Experiment 1. However, when stimuli and effects did not overlap (Experiment 2) the pattern was different. Participants in the forced choice condition showed an influence of action effect compatibility in early blocks which apparently vanished when responding supposedly became more stimulus driven with practice. We assume that action effect compatibility played out earlier in practice in RTs of Experiment 2 as compared to Experiment 1, because the arrows as action effects were (a) salient and (b) were easy to tell apart from the stimuli. Occasional reports by participants in Experiment 1 suggest that it could be somewhat of a challenge not to start responding to the action effects. For instance, non-ambiguous stimuli and action effects could only be held apart by relying on temporal context information (fixation cross, response, presentation time). Placing special emphasis on avoiding to react to the action effects might have led to conflict driven down-weighting of the action effects (compare, e.g., Hommel et al., 2001; Kruschke, 2003). Once that practice with the task might have helped to establish the context to avoid the risk of such confusions, task processing might have become more stimulus based. One can thus speculate that two changes with practice together diminished the impact of action effect compatibility on RT.

Surprisingly, Experiment 1 and 2 differed with respect to speed on ambiguous vs. non-ambiguous trials. Instructions suggested to the participants that difficult to discriminate stimuli could be responded to quickly – either by a spontaneous free choice (free choice condition) or by spontaneously reacting to the impression that the stimulus leaves. Following the suggestion to choose or react quickly and spontaneously, participants in Experiment 2 reacted faster to ambiguous compared to non-ambiguous trials. In Experiment 1, however, ambiguous trials led to slower responses compared to non-ambiguous trials. As action effects and their overlap with the stimuli were the only difference between Experiment 1 and 2, this variation may be responsible for this difference. In Experiment 1, clouds of dots shifted toward one of the borders of the reference frame were presented as action effect in each trial and as stimulus in two thirds of the trials. Clouds in the center of the reference frame were therefore rare compared to clouds close to one of the boarders of the reference frame. This frequency difference may have contributed to the slowing (e.g., Fitts et al., 1963). Furthermore, the analysis of choice data suggested that participants might have faced problems in disentangling stimuli and effects in Experiment 1 (whereas this distinction was clear in Experiment 2). Ambiguous trials in Experiment 1 were special in that they constituted the only case in which the position of the cloud of dots could not be judged with some certainty. The position of the cloud of dots could be discriminated in the non-ambiguous trials and in the action effects. Ironically, a response was therefore required for those events on the screen that were the hardest to categorize.

In order to integrate (a) the effects of instructed action mode and (b) the surprising difference when using clouds of dots vs. arrows as action effects, one might suggest that free choice leads to the anticipation of action effects in situations in which the effects would otherwise be only minimally processed. Conditions of otherwise reduced anticipation of action effects might include cases where effects are of low salience (cf. Janczyk et al., 2012b,c), the choice reaction task is characterized by a simple mapping that should render stimulus based control feasible (cf. Herwig and Waszak, 2009), or a choice reaction task in which stimuli and effects overlap such that action effects might be down-weighted in order to reduce crosstalk (e.g., Experiment 1). With demanding choice reaction tasks and/or salient effects, however, effect anticipation might be substantial irrespective of instructed action mode.

### Debated concepts with behavioral consequences

One could debate the extent to which free choice tasks differ from forced choice tasks in a way that can be described in a theoretically coherent manner (cf. Walter, 2002). However, empirical research suggests that the mode of action implied in forced choice vs. free choice behavior makes a difference at some level of processing as it has consequences for the acquisition/expression of action effect knowledge (cf. Herwig et al., 2007; Herwig and Waszak, 2009; Pfister et al., 2011). Recently Neuringer and Jensen (2010) have bypassed philosophical problems of the free will debate by proposing a behavioral account of operant action that emphasizes the role of variability in action as well as adaptive and task contingent changes in variability of action. However, while the above accounts might be taken to suggest that structure of the task material (i.e., discriminability of stimuli) and variability of action (i.e., variability of key presses) are the dominant constituents of intention based vs. stimulus based action mode, our results suggest a surprisingly constructivist perspective of action control. Apart from the instructions, people in the free choice vs. forced choice condition were confronted with identical task material and showed similar amounts of variability in behavior. Yet, action effect compatibility played out differently in these conditions. One could have doubted the power of the instruction manipulation. One could have expected that irrespective of instructions, the objectively indiscriminable stimuli (ambiguous, centrally placed clouds of dots) would have led to guessing behavior, internally chosen action, and an impact of action effect knowledge on RT and choice. From this perspective, non-ambiguous stimuli (clouds of dots placed close to one of the borders of the frame) should have led to stimulus driven action control, reducing the impact of action effect knowledge on RT and choice. Indeed, irrespective of instruction, stimuli that could be discriminated were responded to according to their spatial stimulus characteristics in the large majority of the trials rather than based on guessing. With respect to the usage of action effect knowledge, however, the impact of instructions on task processing seemed to be stronger than the impact of stimulus characteristics, as the instructions concerning the ambiguous stimuli also influenced performance in trials with non-ambiguous stimuli.

The findings of the current study stress the importance of considering the dynamics of the acquisition of action effect knowledge and the dynamics of action control modes. The results suggest that the mode of action control (stimulus based vs. intention based) can be influenced quickly by the instructions but might be subjected to practice-related changes some of which are slow. While strong in the beginning, the RT effect of action effect compatibility had vanished in the choice reaction condition of Experiment 2 by the end of practice. Therefore, taking a snapshot at a single point in time might have led to the wrong conclusions. Wolfensteller and Ruge (2011) have recently presented an approach including tests of action effect knowledge at different points in time over the course of practice, but did not vary action mode. We extended their results by providing evidence that there might be practice-related changes in how instruction-induced mode of action control influences the impact of action effect associations on behavior.

With its strong emphasis on the impact of instruction on action control (see also Hommel, 1993) the current research shows interesting parallels to the Baldwin–Titchener debate of the end of the nineteenth century (cf. Baldwin, 1895; Titchener, 1895). The debate was on whether or not RTs are regularly shorter when people concentrate on the response rather than on the stimulus. The Wundt camp suggested that this should be the case, at least in well-behaving research participants, as the apperception component was reduced if emphasis was drawn to the response rather than to the stimulus. Baldwin and followers in turn alluded to inter-individual differences, suggesting that some people may be faster in the stimulus based mode and others in the response based mode. Probably, the most interesting point about the debate was one that was not at the focus of the discussion. Either camp, the functionalists around Baldwin as well as the structuralists from the Wundt lab, heavily relied on the power of instruction. They instructed participants (often the researchers themselves) to adhere to an action mode relevant to the experiment: that is to concentrate on the response vs. to concentrate on the stimulus.

In conclusion, the present experiments suggest that instructions can determine the mode of action control and by this the impact of action effect associations on behavior. Predictable action effects influenced RT and response choice differently, depending on whether instructions had suggested free choice vs. forced choice for ambiguous stimuli. The instruction effect transferred to non-ambiguous stimuli. This indicates that instructions rather than stimulus discriminability determined the extent to which action effects were weighted relative to the stimuli.

## Conflict of Interest Statement

The authors declare that the research was conducted in the absence of any commercial or financial relationships that could be construed as a potential conflict of interest.

## References

[B1] Abrahamse E. L. Jiménez J. Verwey W. B. Clegg B. A. (2010). Representing serial action and perception. Psychon. Bull. Rev. 17, 603–623 10.3758/PBR.17.5.603 21037157

[B2] Ackerman P. L. Woltz D. J. (1994). Determinants of learning and performance in an associative memory substitution task: task constraints, individual differences, volition, and motivation. J. Educ. Psychol. 86, 487–515 10.1037/0022-0663.86.1.150

[B3] Ansorge U. (2002). Spatial intention-response compatibility. Acta Psychol. (Amst.) 109, 285–299 10.1016/S0001-6918(01)00062-2 11881904

[B4] Baldwin J. M. (1895). Types of reaction. Psychol. Rev. 2, 259–273 10.1037/h0074743

[B5] Band G. P. H. van Steenbergen H. Ridderinkhof K. R. Falkenstein M. Hommel B. (2009). Action-effect negativity: irrelevant action effects are monitored like relevant feedback. Biol. Psychol. 82, 211–218 10.1016/j.biopsycho.2009.06.011 19665516

[B6] Brass M. Bekkering H. Wohlschläger A. Prinz W. (2000). Compatibility between observed and executed finger movements: comparing symbolic, spatial, and imitative cues. Brain Cogn. 44, 124–143 10.1006/brcg.2000.1225 11041986

[B7] Dreisbach G. Fischer R. (2011). If it’s hard to read… try harder! Processing fluency as signal for effort adjustments. Psychol. Res. 75, 376–383 10.1007/s00426-010-0319-y 21210144

[B8] Elsner B. Hommel B. (2001). Effect anticipation and action control. J. Exp. Psychol. Hum. Percept. Perform. 27, 229–240 10.1037/0096-1523.27.1.229 11248937

[B9] Fitts P. M. Peterson J. R. Wolpe G. (1963). Cognitive aspects of information processing: II. Adjustments to stimulus redundancy. J. Exp. Psychol. 65, 423–432 10.1037/h0047993 13945344

[B10] Gaschler R. Frensch P. A. Cohen A. Wenke D. (2012). Implicit sequence learning based on instructed task set. J. Exp. Psychol. Learn. Mem. Cogn. 38, 1389–1407 10.1037/a0028071 22545612

[B11] Greenwald A. G. (1970). A choice reaction time test of ideomotor theory. J. Exp. Psychol. 86, 20–25 10.1037/h0029960 5482033

[B12] Hazeltine E. (2002). “The representational nature of sequence learning: evidence for goal-based codes,” in Attention and Performance, Vol. 19, eds Prinz W. Hommel B. (Oxford: University Press), 673–689

[B13] Herwig A. Prinz W. Waszak F. (2007). Two modes of sensorimotor integration intention-based and stimulus-based actions. Q. J. Exp. Psychol. 60, 1540–1554 10.1080/17470210601119134 17853217

[B14] Herwig A. Waszak F. (2009). Intention and attention in ideomotor learning. Q. J. Exp. Psychol. 62, 219–227 10.1080/17470210802373290 18932052

[B15] Herwig A. Waszak F. (2012). Action-effect bindings and ideomotor learning in intention- and stimulus-based actions. Front. Psychol. 3:444 10.3389/fpsyg.2012.00444 23112785 PMC3481004

[B16] Hoffmann J. Lenhard A. Sebald A. Pfister R. (2009). Movements or targets: what makes an action in action-effect learning? Q. J. Exp. Psychol. 62, 2433–2449 10.1080/17470210902922079 19526438

[B17] Hommel B. (1993). Inverting the Simon effect by intention. Psychol. Res. 55, 270–279 10.1007/BF00419608 8416040

[B18] Hommel B. (1996). The cognitive representation of action: automatic integration of perceived action effects. Psychol. Res. 59, 176–186 10.1007/BF00425832 8923816

[B19] Hommel B. (2000). “Intentional control of automatic stimulus-response translation,” in Interaction Between Dissociable Conscious and Non-Conscious Processes, eds Rossetti Y. Revonsuo A. (Amsterdam: John Benjamins Publishing Company), 223–244

[B20] Hommel B. Alonso D. Fuentes L. J. (2003). Acquisition and generalization of action effects. Vis. Cogn. 10, 965–986 10.1080/13506280344000176

[B21] Hommel B. Müsseler J. Aschersleben G. Prinz W. (2001). The theory of event coding (TEC): a framework for perception and action planning. Behav. Brain Sci. 24, 849–937 10.1017/S0140525X01000103 12239891

[B22] Jahanshahi M. Saleem T. Ho A. K. Dirnberger G. Fuller R. (2006). Random number generation as an index of controlled processing. Neuropsychology 20, 391–399 10.1037/0894-4105.20.4.391 16846257

[B23] James W. (1890). The Principles of Psychology. New York: Dover Publications

[B24] Janczyk M. Heinemann A. Pfister R. (2012a). Instant attraction: immediate action-effect bindings occur for both, stimulus- and goal-driven actions. Front. Psychol. 3:446 10.3389/fpsyg.2012.00446 23112787 PMC3481005

[B25] Janczyk M. Pfister R. Crognale M. A. Kunde W. (2012b). Effective rotations: action-effects determine the interplay of mental and manual rotations. J. Exp. Psychol. Gen. 141, 489–501 10.1037/a0026997 22268853

[B26] Janczyk M. Pfister R. Kunde W. (2012c). On the persistence of tool-based compatibility effects. J. Psychol. 220, 16–22

[B27] Kemper M. Umbach V. J. Schwager S. Gaschler R. Frensch P. A. Stürmer B. (2012). What I say is what I get: stronger effects of self-generated vs. cue-induced expectations in event-related potentials. Front. Psychol. 3:562 10.3389/fpsyg.2012.00562 23403896 PMC3565970

[B28] Kruschke J. K. (2003). Attention in learning. Curr. Dir. Psychol. Sci. 12, 171–175 10.1111/1467-8721.01254

[B29] Kubovy M. Holcombe A. O. Wagemans J. (1998). On the lawfulness of grouping by proximity. Cogn. Psychol. 35, 71–98 10.1006/cogp.1997.0673 9520318

[B30] Kubovy M. Van den Berg M. (2008). The whole is equal to the sum of its parts: a probabilistic model of grouping by proximity and similarity in regular patterns. Psychol. Rev. 115, 131–154 10.1037/0033-295X.115.1.131 18211188

[B31] Kunde W. (2001). Response-effect compatibility in manual choice reaction tasks. J. Exp. Psychol. Hum. Percept. Perform. 27, 387–394 10.1037/0096-1523.27.2.387 11318054

[B32] Kunde W. Koch I. Hoffmann J. (2004). Anticipated action effects affect the selection, initiation, and execution of actions. Q. J. Exp. Psychol. A 57, 87–106 14681005 10.1080/02724980343000143

[B33] Maes J. H. (2006). Response bias induces in rats by response effects. Q. J. Exp. Psychol. 59, 1346–1356 10.1080/17470210600639033 16846965

[B34] Nattkemper D. Ziessler M. Frensch P. A. (2010). Binding in voluntary action control. Neurosci. Biobehav. Rev. 34, 1092–1101 10.1016/j.neubiorev.2009.12.013 20036685

[B35] Neuringer A. Jensen G. (2010). Operant variability and voluntary action. Psychol. Rev. 117, 972–993 10.1037/a0019499 20658860

[B36] Pfister R. Kiesel A. Hoffmann J. (2011). Learning at any rate: action-effect learning for stimulus-based actions. Psychol. Res. 75, 61–65 10.1007/s00426-010-0288-1 20490862

[B37] Pfister R. Kiesel A. Melcher T. (2010). Adaptive control of ideomotor effect anticipations. Acta Psychol. (Amst.) 135, 316–322 10.1016/j.actpsy.2010.08.006 20875631

[B38] Prinz W. (1987). “Ideo-motor action,” in Perspectives on Perception and Action, eds Heuer H. Sanders A. F. (Hillsdale: Erlbaum), 47–76

[B39] Shin Y. K. Proctor R. W. Capaldi E. J. (2010). A review of contemporary ideomotor theory. Psychol. Bull. 136, 943–974 10.1037/a0021628 20822210

[B40] Titchener E. B. (1895). The type-theory of simple reaction. Mind 4, 506–514 10.1093/mind/IV.16.506

[B41] Umbach V. J. Schwager S. Frensch P. A. Gaschler R. (2012). Does explicit expectation really affect preparation? Front. Psychol. 3:378 10.3389/fpsyg.2012.00378 23248606 PMC3521289

[B42] Walter H. (2002). “The neurophilosophy of free will,” in The Oxford Handbook of Free Will, ed. Kane R. (Oxford: Oxford University Press), 565–576

[B43] Willingham D. B. Wells L. A. Farrell J. M. Stemwedel M. E. (2000). Implicit motor sequence learning is represented in response locations. Mem. Cognit. 28, 366–375 10.3758/BF03198552 10881554

[B44] Wolfensteller U. Ruge H. (2011). On the timescale of stimulus-based action-effect learning. Q. J. Exp. Psychol. 64, 1273–1289 10.1080/17470218.2010.546417 21416458

[B45] Ziessler M. Nattkemper D. (2001). Learning of event sequences is based on response-effect learning: further evidence from a serial reaction task. J. Exp. Psychol. Learn. Mem. Cogn. 27, 595–613 10.1037/0278-7393.27.3.595 11394669

[B46] Ziessler M. Nattkemper D. Frensch P. A. (2004). The role of anticipation and intention for the learning of effects of self-performed actions. Psychol. Res. 68, 163–175 10.1007/s00426-003-0153-6 14634810

